# Metabolomic Profiling of Disease Progression Following Radiotherapy for Breast Cancer

**DOI:** 10.3390/cancers17050891

**Published:** 2025-03-05

**Authors:** Alexandra N. McMahon, Isildinha M. Reis, Cristiane Takita, Jean L. Wright, Jennifer J. Hu

**Affiliations:** 1 Department of Public Health Sciences, University of Miami Miller School of Medicine, Miami, FL 33136, USA; axm8143@miami.edu (A.N.M.); ireis@med.miami.edu (I.M.R.); 2 Sylvester Comprehensive Cancer Center, University of Miami Miller School of Medicine, Miami, FL 33136, USA; ctakita@med.miami.edu; 3 Department of Radiation Oncology, University of Miami Miller School of Medicine, Miami, FL 33136, USA; 4 Department of Radiation Oncology, University of North Carolina School of Medicine, Chapel Hill, NC 27514, USA; jean_wright@med.unc.edu

**Keywords:** breast cancer, metabolomics, biomarkers, pathways, prognosis

## Abstract

Background: This study aims to explore metabolic biomarkers and pathways in breast cancer prognosis. Methods: We performed a global post-radiotherapy (RT) urinary metabolomic analysis of 120 breast cancer patients: 60 progression-free (PF) patients as the reference and 60 with progressive disease (PD: recurrence, second primary, metastasis, or death). UPLC-MS/MS (Metabolon Inc.) identified 1742 biochemicals (1258 known and 484 unknown structures). Following normalization to osmolality, log transformation, and imputation of missing values, a Welch’s two-sample *t*-test was used to identify biochemicals and metabolic pathways that differed between PF and PD groups. Data analysis and visualization were performed with MetaboAnalyst. Results: Metabolic biomarkers and pathways that significantly differed between the PD and PF groups were the following: amino acid metabolism, including phenylalanine, tyrosine, and tryptophan biosynthesis (impact value (IV) = 1.00; *p* = 0.0007); histidine metabolism (IV = 0.60; *p* < 0.0001); and arginine and proline metabolism (IV = 0.70; *p* = 0.0035). Metabolites of carbohydrate metabolism, including glucose (*p* = 0.0197), sedoheptulose (*p* = 0.0115), and carboxymethyl lysine (*p* = 0.0098), were elevated in patients with PD. Gamma-glutamyl amino acids, myo-inositol, and oxidative stress biomarkers, including 7-Hydroxyindole Sulfate and sulfate, were elevated in patients who died (*p* ≤ 0.05). Conclusions: Amino acid metabolism emerged as a key pathway in breast cancer progression, while carbohydrate and oxidative stress metabolites also showed potential utility as biomarkers for breast cancer progression. These findings demonstrate applications of metabolomics in identifying metabolic biomarkers and pathways as potential targets for predicting breast cancer progression.

## Simple Summary

This study investigated urinary metabolomic profiles post radiotherapy of 120 breast cancer patients; half experienced disease progression (recurrence, metastasis, or death) and half remained progression-free. Using ultrahigh-performance liquid chromatography-tandem mass spectrometry (UPLC-MS/MS), 1742 metabolites were identified and analyzed with MetaboAnalyst. Significant differences were observed in amino acid metabolism, including phenylalanine, tyrosine, and tryptophan biosynthesis (impact value (IV) = 1.00; *p* = 0.0007), histidine metabolism (IV = 0.60; *p* < 0.0001), and arginine and proline metabolism (IV = 0.70; *p* = 0.0035). This study suggests that amino acid metabolism, in addition to metabolites of carbohydrate metabolism and oxidative stress, may be associated with breast cancer progression.

## 1. Introduction

Breast cancer is the most frequently diagnosed cancer and the second leading cause of cancer death in American women, with over 313,510 new cases, 42,780 deaths, and 4 million people living as breast cancer survivors in 2024 [1]. Recent advances in screening and treatment strategies and the aging population in the United States have led to a growing population of breast cancer survivors [2]. Yet, this disease does not affect all patient populations equally [3], and there is growing evidence to suggest molecular signatures, tumor heterogeneity, and genetic variations may contribute to tumor progression and patient prognosis [4,5]. Investigation of these metabolic risk factors has the potential to improve prognosis and quality of life for survivors while pointing researchers toward more effective and targeted treatment options.

Several studies have found associations between various metabolic signatures, breast cancer subtypes, and prognosis [6,7,8,9]. In fact, specific metabolic profiles have been associated with more aggressive breast cancer subtypes, which may consequently affect clinical outcomes and treatment strategies [10]. Multiple studies have also identified associations between metabolic signatures and treatment response in breast cancer patients [11]. Growing evidence suggests that the metabolic alterations in breast cancer are influenced by both tumor intrinsic factors and the tumor microenvironment [12], highlighting the complex interplay of metabolic reprogramming in cancer progression. Specifically, alterations in amino acid metabolism, lipid metabolism, and glycolysis-related pathways have been associated with breast cancer progression [13]. In response, researchers have shown increased interest in metabolic reprogramming in cancer, exploring the relationship between metabolic pathways and cancer prognosis to potentially provide the framework for the development of novel therapeutics and precision medicine targets.

The field of metabolomics has emerged as a result—using the large-scale study of small metabolites to comprehensively analyze metabolic processes in biological samples from patient populations. The metabolome, reflecting downstream processes of gene expression and protein activity, offers critical insight into the phenotype and disease state of various conditions, including cancer [14]. With growing interest in the role of metabolic pathways in cancer, applications of metabolomics in cancers, including breast, have become increasingly widespread [15]. Recent advances in mass spectrometry and imaging techniques have further broadened the applications of metabolomics in identifying and validating precision biomarkers, and the results from several recent studies have shown applications of metabolomics in breast cancer diagnosis and subtype analysis, characterization of heterogeneity of breast cancer, and prognosis [16,17,18,19,20]. Among metabolomic approaches, urine metabolomics offers several benefits, including its non-invasive collection and its ability to capture systemic metabolic changes for various cancers [21,22].

Despite these recent advancements, there remains a dearth of literature exploring metabolite biomarkers in breast cancer prognosis. Therefore, in the present study, we performed global metabolomic profiling on urine samples from 120 breast cancer patients to explore variations in metabolite biomarkers associated with disease progression. This study aims to identify potential predictive biomarkers and pathways by comparing metabolic profiles of patients who experienced progressive disease events, including recurrence, second primary, metastasis, or death, to patients without progressive disease.

## 2. Materials and Methods

### 2.1. Study Population

Patients were recruited from 2008 to 2014 from the University of Miami Sylvester Comprehensive Cancer Center (SCCC) and Jackson Memorial Hospital (JMH), in Florida, United States. Eligible participants included women newly diagnosed with breast cancer (stages 0–III) aged 18 years or older, who had undergone breast-conserving surgery, and were scheduled to receive adjuvant radiotherapy (RT) to the whole breast with or without regional lymph nodes (total dose ≥ 40 Gy, dose per fraction ≥ 2.0 Gy). Patients receiving concurrent chemotherapy with RT were excluded. This study adhered to guidelines provided by the University of Miami’s Institutional Review Board, and written informed consent was obtained from all participants after providing a comprehensive explanation of the protocol in either English or Spanish. Participants for this analysis, 60 patients with progressive disease (case: recurrence, second primary, metastasis, or death) and 60 patients without events at the last follow-up visit, were selected from a prior parent study [23] by implementation of a frequency-matching study design with matches based on demographics and tumor and clinical characteristics.

### 2.2. Metabolomic Profiling

Urine samples were collected immediately after the completion of RT and stored at −80 °C. The samples were processed at Metabolon, Inc. (Durham, NC, USA) using ultrahigh-performance liquid chromatography-tandem mass spectroscopy (UPLC-MS/MS), identifying a total of 1742 biochemicals (1258 known and 484 unknown structures). Samples were prepared using the MicroLab STAR^®^ system with recovery standards added before the extractions for quality control (QC). Organic and aqueous extractions were performed to remove the protein fraction while preserving small molecules. The extract was separated into five fractions: one for reverse phase (RP) UPLC-MS/MS analysis with negative ion mode electrospray ionization (ESI), another for HILIC/UPLC-MS/MS analysis with negative ion mode ESI, and two for distinct RP/UPLC-MS/MS analyses using positive ion mode ESI. A TurboVap^®^ vortex was used to evaporate the organic solvent.

The raw data outputs were archived, extracted, and accessioned into the Metabolon Library Information Management System (LIMS), which included components for data extraction, peak identification, QC, and compound identification. Normalization steps were achieved to correct inter-day tuning differences between instruments employed in multiple-day studies. Before statistical analyses, the dataset was normalized to osmolality, and log transformations were performed, with median scaling and imputation for missing values using the minimum observed value for each compound.

### 2.3. Statistical Analysis

Welch’s two-sample *t*-test was used to identify statistically significant biochemical fold-change differences between breast cancer patients with progressive disease event (recurrence, second primary cancer, metastasis, or death) (group PD) and event-free breast cancer patients (group PF, the reference). Data from two patients, one from the PF and one PD group, were identified as outliers and were excluded from statistical contrast comparisons. Pathway enrichment and topology analyses were performed using MetaboAnalyst 6.0. An exploratory *p*-value threshold of 0.5 was used to identify metabolites for pathway analysis. Metabolites that varied between patients with PD and PF patients (*p* < 0.5) were uploaded for pathway analysis in MetaboAnalyst. All other analyses were performed using SAS v. 9.4 (SAS Inc., Cary, NC, USA).

## 3. Results

Table 1 summarizes the patient characteristics of the study population. Due to the implemented frequency-matching study design, the PF and PD groups were similar with respect to demographics and tumor and clinical characteristics. The average age at breast cancer diagnosis was 54.9 years, and most patients were Hispanic white (63%), followed by non-Hispanic white/other (26%), and African American (12%). Among all patients, 38% were obese, 36% were overweight, and 26% were normal weight. At breast cancer diagnosis, 58% of patients were clinical tumor stages III–IV and the remaining 42% of patients were stages 0–II. Most of the population was estrogen receptor (ER)-positive (70%), 53% were progesterone receptor (PR)-positive, 78% were human epidermal growth factor receptor 2 (HER2) negative, and 25% were triple-negative. PF patients were event-free for a follow-up median of 6.3 years (Range: 2.2–9.8). Event status in the PD group included 10 (17%) recurrences, 39 (65%) metastases, and 34 (28%) deaths. Time-to-event median was 2.4 years (range: 0.0–8.7).

Table 2 illustrates the top 30 metabolites with variations by event status compared to the reference PF group. Significant differences (*p* ≤ 0.05 from Welch’s two-sample *t*-tests) were observed for metabolites of carbohydrate metabolism, including glucose, with a 3.22-fold change observed in patients with progressive disease, sedoheptulose (1.30-fold), and carboxymethyl lysine (1.29-fold). Conversely, in patients with recurrence, glucose was lower than the reference group (0.71-fold). Myo-inositol levels were elevated in patients who died (1.57-fold). Further, metabolites related to amino acid metabolism, including gamma-glutamyl isoleucine and gamma-glutamyl valine, were elevated in patients who died (1.19-fold and 1.15-fold, respectively; *p* < 0.05) and those who experienced metastasis (1.15-fold and 1.16-fold, respectively). Additionally, N-alpha-acetylornithine, associated with lysine degradation, was higher in patients who died (1.50, *p* ≤ 0.01). Metabolites related to oxidative stress including 7-hydroxyindole sulfate and sulfate were elevated in patients who died (1.74 and 1.13, *p* ≤ 0.05) and those with metastasis (1.58 and 1.12, *p* ≤ 0.05). Dopamine 4-sulfate was also higher in patients who experienced metastasis (2.17, *p* ≤ 0.01). Saccharin, related to lipid metabolism, was higher in patients with metastasis (9.37, *p* ≤ 0.01). 2,3-dimethylsuccinate, associated with the TCA cycle, was lower in patients who died and those with metastasis (0.59 and 0.71, respectively; *p* ≤ 0.05). Box plots presenting the distribution of these top metabolites by event status are shown in Supplementary Figure S1.

Table 3 lists the top metabolic pathways associated with breast cancer progression. Phenylalanine, tyrosine, and tryptophan biosynthesis was the major pathway associated with disease progression reaching maximum pathway importance (impact value IV = 1.0) and high significance (*p* = 0.0007). Following, phenylalanine metabolism was the second pathway of interest due to its high impact value and significance (IV = 0.86; *p* = 0.0194). Other significant pathways include histidine (IV = 0.60; *p* < 0.0001), beta-alanine (IV = 0.62; *p* = 0.0015), and arginine and proline metabolism (IV = 0.70; *p* = 0.0034).

Table 4 summarizes the prognostic significance, biological mechanisms, and potential therapeutic targets of top metabolic pathways identified in this study. Further, it lists the matched metabolites from the pathway analysis and their node importance (calculated by relative betweenness centrality) which are used to calculate the pathway impact value. The pathway phenylalanine, tyrosine, and tryptophan biosynthesis had maximum importance (impact value of 1), with L-Phenylalanine and L-Tyrosine metabolites emerging as the key metabolites (node importance = 0.50).

## 4. Discussion

As breast cancer continues to burden patient populations globally, understanding the biological predictors of patient prognosis is critical. Growing evidence suggests that variations in metabolic profiles may play a role in the disease progression and treatment outcomes for various cancers [38], yet our knowledge regarding metabolite biomarkers’ relation to patient outcomes for breast cancer remains limited. Therefore, in this study, metabolomic profiles of 120 breast cancer patients with and without progressive disease were explored. By capitalizing on advancements in metabolomics, our findings explore the biological mechanisms underlying breast cancer prognosis, which may lead to further advances improving breast cancer treatments and patient outcomes. To the best of our knowledge, this is one of the first global metabolomics studies to evaluate metabolic pathways and biomarkers in breast cancer prognosis.

The findings from the current study identified several metabolic pathways that significantly differed among patients with and without progressive disease. Amino acid metabolism emerged as a key pathway of interest. As amino acids are pivotal in energy metabolism and protein synthesis, they may also be critical in breast cancer progression [39]. Previous findings have suggested that disturbances in amino acid metabolism, including glutamine and branch-chain amino acids (BCAAs), can affect tumor growth and survival [40,41]. Increased expression of BCAT1 has been involved in promoting the development of breast cancer [42]. Further, our findings are consistent with past research suggesting the potential prognostic significance of elevated levels of gamma-glutamyl amino acids in breast cancer [43]. Our interest in amino acid metabolism in breast cancer prognosis was furthered by the pathway analysis revealing that most of the high-impact pathways were related to amino acid metabolism.

Oxidative stress and citrate cycle (TCA cycle) metabolites, closely linked to amino acid metabolism, emerged as other key metabolites of interest in our study. Past studies have observed disturbances in oxidative stress-related metabolites in breast cancer patient populations [44,45]. The findings from the current study contribute to a growing body of evidence suggesting that oxidative stress and chronic inflammation may be associated with poorer prognosis in breast cancer [46]. Similarly, our TCA cycle-related findings align with past research suggesting that disruptions in the TCA cycle can promote altered energy metabolism, leading to tumor growth and survival [13]. Collectively, this study’s findings highlight the role of metabolic dysregulation and the interplay of amino acids, oxidative stress, and the TCA cycle in breast cancer progression, suggesting the potential utility of these metabolites as precision medicine targets for breast cancer. The observed alterations in amino acid metabolism, oxidative stress, and TCA cycle pathways align with prior findings linking metabolic reprogramming to tumor progression and more aggressive breast cancer subtypes, suggesting that these pathways may have clinical utility for risk stratification and targeted therapies in breast cancer [13].

Lastly, carbohydrate metabolism, an area of growing interest in breast cancer research, emerged as another pathway of interest. Glucose levels were significantly higher in most patients with PD. Increased glucose excretion in these patients suggests a hyperglycemic state, further supported by the elevation of advanced glycation end-product metabolite carboxymethyl lysine. Sedoheptulose, a pentose phosphate pathway-related metabolite, was also significantly higher in several event groups compared to the reference group. Our findings contribute to the growing body of evidence suggesting the importance of glucose in breast cancer metabolism and prognosis [47]. Carbohydrate metabolism supports tumor growth, increased energy demands, and adaptation to a hypoxic microenvironment, processes linked to tumorigenesis and disease progression in breast cancer [48]. A number of studies have shown a potential association between increased glucose metabolism and breast cancer prognosis [47,49], and elevated excretion of glucose has been associated with chemoresistance, cell proliferation, metastasis, and mortality in breast cancer [50,51]. As glutaminase inhibitors have recently become clinically available for a variety of cancers and chronic conditions [52,53,54,55], study findings encourage further exploration of these inhibitors as potential therapies targeting metabolic flexibility, including glucose metabolism, in breast cancer.

The present study had several strengths and limitations. Strengths include our use of biological samples and clinical data from a prospective cohort, capturing a highly diverse breast cancer patient population. This study leveraged advances in metabolomics-based technologies, utilizing liquid chromatography-mass spectrometry (LC-MS) to analyze biological compounds associated with breast cancer progression. The integration of LC-MS has enhanced metabolic profiling by improving compound profiling capacities and enhancing sensitivity and selectivity. A limitation of this research was the relatively moderate sample size (n = 120) and lack of other confounding factors. To overcome this limitation to some extent, we employed a frequency-matched study design to select samples (60 PD and 60 PF). Additionally, although samples were stored at −80 °C, potential metabolite degradation over time is possible. Future research should focus on expanding cohort sizes, incorporating multi-omic approaches, and conducting metabolomics assays at the beginning of longitudinal studies to better characterize metabolic alterations in predicting breast cancer disease progression. As some of the metabolomic differences observed between the two groups were subtle, additional longitudinal analyses with larger sample sizes, comprehensive clinical data, lifestyle factors, and other comorbidities are critical to validate our findings and further explore applications of metabolomics in breast cancer prognosis.

## 5. Conclusions

In the present study of breast cancer patients receiving adjuvant RT, we identified several post-RT metabolic biomarkers and pathways that may contribute to breast cancer progression. Specifically, alterations in amino acid metabolism, carbohydrate metabolism, and oxidative stress-related metabolites were observed in patients who exhibited a greater likelihood of disease progression. With increasing interest in targeting tumor metabolism in precision medicine and our data suggesting multiple metabolic pathways in breast cancer progression, future research is warranted to further evaluate the application of metabolomics in breast cancer progression and targeted precision interventions.

## Figures and Tables

**Table 1:** Patient clinical and demographic characteristics.

Variables	N	%	PF	%	PD	%	*p*-Value
Total	120	100%	60	100%	60	100%	
Age at Diagnosis							0.70
<50	42	35%	20	33%	22	37%	
≥50	78	65%	40	67%	38	63%	
Mean (SD)	54.9 (10.9)		55.2 (10.5)		54.6 (11.4)		0.78
Race							0.52
AA	14	12%	9	15%	5	8%	
HW	75	63%	36	60%	39	65%	
NHW/Other	31	26%	15	25%	16	27%	
BMI							0.72
Normal	31	26%	14	23%	17	28%	
Overweight	43	36%	21	35%	22	37%	
Obese	46	38%	25	42%	21	35%	
Clinical Tumor Stage							1.0
0–II	50	42%	25	42%	25	42%	
III–IV	70	58%	35	58%	35	58%	
Triple negative							1.0
No	90	75%	45	75%	45	75%	
Yes	30	25%	15	25%	15	25%	
ER							0.43
Negative	36	30%	20	33%	16	27%	
Positive	84	70%	40	67%	44	73%	
PR							0.71
Negative	56	47%	29	48%	27	45%	
Positive	64	53%	31	52%	33	55%	
HER2							0.84
Missing	5	4%	3	5%	2	3%	
Negative	94	78%	47	78%	47	78%	
Positive	21	18%	10	17%	11	18%	
Event status							NA
No event	NA	NA	60	100%	NA	NA	
Recurrence	NA	NA	NA	NA	10	17%	
Metastasis	NA	NA	NA	NA	39	65%	
Death	NA	NA	NA	NA	34	28%	
Follow-up Time (years)							NA
Median (Range)	5.2 (0.0–9.8)		6.3 (2.2–9.8)		2.4 (0.0–8.7)		

Abbreviations: PF: progression free, PD: progressive disease, AA: black or African American, HW: Hispanic white, NHW: non-Hispanic white, BMI: body mass index, ER: estrogen receptor, PR: progesterone receptor, HER2: human epidermal growth factor receptor, SD: standard deviation, NA: not applicable. *p*-values from chi-square or Fisher’s exact tests for categorical variables and from *t*-tests for continuous variables. Note: no group difference due to frequency-matching study design.

**Table 2:** Differences (fold-change) of metabolites between PD group, or a subset given by event status in PD group, compared to the reference PF group.

Biochemical Name	All Patients with PD	*p* ^a^	Death (n = 36)	Recurrence (n = 10)	Metastasis (n = 40)	Second Primary (n = 7)
7-Hydroxyindole Sulfate	1.73	0.0022	1.74 **	1.32 *	1.58 **	1.72
Gamma-glutamylisoleucine	1.21	0.0045	1.19 *	1.00	1.15 *	1.14
Sulfate	1.17	0.0054	1.13 *	1.00	1.12 *	1.06
Myo-inositol	1.50	0.0082	1.57 *	1.47	1.21	0.76
Gamma-glutamylvaline	1.17	0.0095	1.15 *	0.99	1.16 *	1.09
N^6^-Carboxymethyllysine	1.29	0.0098	1.24	0.93	1.21	1.27
Sedoheptulose	1.30	0.0115	1.09	1.01	1.41 *	0.81
Gamma-glutamylthreonine	1.25	0.0127	1.25	1.09	1.21	1.42 *
N-alpha-acetylornithine	1.39	0.0142	1.50 **	0.95	1.25	1.14
Dopamine 4-sulfate	1.75	0.0146	1.24	1.95	2.17 **	1.35
3,4-dihydroxybutyrate	1.39	0.0155	1.13	1.05	1.46	1.18
Saccharin	6.92	0.0155	2.79	1.19	9.37 **	3.52
Homocitrulline	1.31	0.0156	1.06	1.18	1.33	1.27
2,3-Dimethylsuccinate	0.70	0.0166	0.59 **	0.99	0.71 *	0.92
Glycine conjugate of C_6_H_10_O_2_	1.44	0.0171	1.62 *	1.64	1.28	1.84
N^1^-Methyl-2-pyridone-5-carboxamide	1.22	0.0189	1.13	0.92	1.21	1.41 *
Fructosyllysine	1.22	0.0194	1.30 *	1.09	1.10	0.87
Glucose	3.22	0.0197	4.83 *	0.71 *	3.04	0.78
Gamma-glutamylphenylalanine	1.27	0.0202	1.33	1.03	1.25	1.10
Adenosine 5′-monophosphate (AMP)	2.06	0.0204	2.55 *	2.44	2.36	0.78
N-acetylhomocitrulline	1.53	0.0216	1.25	1.05	1.63	1.09
Alpha-hydroxyisocaproate	1.92	0.0217	1.79 *	4.12	1.42	0.84
Carboxymethylarginine	1.33	0.0237	1.21	0.80	1.35	1.21
N-acetylcitrulline	1.30	0.0239	1.29 *	1.11	1.29 *	0.96
N-acetylmethionine sulfoxide	1.25	0.0240	1.21 *	0.94	1.24	1.10
Daidzein	4.86	0.0254	6.84	1.88	7.49 *	20.57
1-palmitoyl-2-linoleoyl-GPC (16:0/18:2)	1.62	0.0257	2.00 *	3.09	1.68	0.78
Cysteine s-sulfate	1.23	0.0272	1.27 *	1.03	1.28 **	1.26
N,N-dimethylalanine	1.54	0.0278	1.46	1.04	1.59 *	1.12
Gamma-glutamylglycine	1.37	0.0282	1.47	1.23	1.30	1.21

Values indicate a ratio compared to the reference PF group. ^a^ *p*-value from the Welch’s two-sample *t*-tests; all *p*-values < 0.05 comparing patients with events (PD group) versus reference PF group. Significant values according to Welch’s *t*-test, comparing a subset of the PD group versus PF group are bolded and underlined, * *p* ≤ 0.05, ** *p* ≤ 0.01.

**Table 3:** Metabolic pathways that differ by breast cancer progression.

Significant Pathways	Total	Expected	Hits	Raw *p* ^a^	−log(*p*)	Holm-Adjusted *p*	FDR *p*	Impact ^b^
Histidine metabolism	16	1.25	9	5.83 × 10^−7^	6.23 × 10^0^	4.67 × 10^−5^	4.67 × 10^−5^	0.60
Arginine biosynthesis	14	1.09	8	2.24 × 10^−6^	5.65 × 10^0^	1.77 × 10^−4^	8.95 × 10^−5^	0.48
Phenylalanine, tyrosine and tryptophan biosynthesis	4	0.31	4	3.55 × 10^−5^	4.45 × 10^0^	2.74 × 10^−3^	7.11 × 10^−4^	1.00
Beta-Alanine metabolism	21	1.64	8	9.47 × 10^−5^	4.02 × 10^0^	7.20 × 10^−3^	1.52 × 10^−3^	0.62
Arginine and proline metabolism	36	2.81	10	2.60 × 10^−4^	3.59 × 10^0^	1.95 × 10^−2^	3.46 × 10^−3^	0.70
Pantothenate and CoA biosynthesis	20	1.56	7	4.92 × 10^−4^	3.31 × 10^0^	3.64 × 10^−2^	5.62 × 10^−3^	0.12
Phenylalanine metabolism	8	0.62	4	1.94 × 10^−3^	2.71 × 10^0^	1.42 × 10^−1^	1.94 × 10^−2^	0.86
Tryptophan metabolism	41	3.20	9	3.31 × 10^−3^	2.48 × 10^0^	2.38 × 10^−1^	2.84 × 10^−2^	0.41
Galactose metabolism	27	2.11	7	3.54 × 10^−3^	2.45 × 10^0^	2.52 × 10^−1^	2.84 × 10^−2^	0.25
Nicotinate and nicotinamide metabolism	15	1.17	5	4.25 × 10^−3^	2.37 × 10^0^	2.98 × 10^−1^	3.09 × 10^−2^	0.19
Citrate cycle (TCA cycle)	20	1.56	5	1.60 × 10^−2^	1.80 × 10^0^	1.00 × 10^0^	1.07 × 10^−1^	0.22
Alanine, aspartate and glutamate metabolism	28	2.19	6	1.81 × 10^−2^	1.74 × 10^0^	1.00 × 10^0^	1.11 × 10^−1^	0.30
Lysine Degradation	30	2.34	6	2.51 × 10^−2^	1.60 × 10^0^	1.00 × 10^0^	1.31 × 10^−1^	0.12
Vitamin B6 metabolism	9	0.70	3	2.76 × 10^−2^	1.56 × 10^0^	1.00 × 10^0^	1.31 × 10^−1^	0.57
Ascorbate and aldarate metabolism	9	0.70	3	2.76 × 10^−2^	1.56 × 10^0^	1.00 × 10^0^	1.31 × 10^−1^	0.76
Pyrimidine metabolism	39	3.05	7	2.79 × 10^−2^	1.55 × 10^0^	1.00 × 10^0^	1.31 × 10^−1^	0.22
Valine, leucine and isoleucine biosynthesis	40	3.12	7	3.17 × 10^−2^	1.50 × 10^0^	1.00 × 10^0^	1.41 × 10^−1^	0.06
Caffeine metabolism	10	0.78	3	3.72 × 10^−2^	1.43 × 10^0^	1.00 × 10^0^	1.56 × 10^−1^	0.69
Tyrosine metabolism	42	3.28	7	4.03 × 10^−2^	1.39 × 10^0^	1.00 × 10^0^	1.61 × 10^−1^	0.40

Total, number of compounds in pathway; hits, matched number of compounds from uploaded data; ^a^, *p*-value from pathway enrichment analysis presenting pathways with raw *p* < 0.05; ^b^, pathway topology analysis impact value, which ranges from 0 to 1 (maximum pathway importance). Abbreviations: FDR, false discovery rate. Note: An exploratory *p*-value threshold of 0.5 was used to identify metabolites for pathway analysis.

**Table 4:** Metabolites and pathways in cancer progression and therapeutic targets.

Pathways (IV)	Matched Metabolites	Node Importance ^a^	Cancer Prognosis	Biological Mechanisms	Therapeutic Targets
Phenylalanine, tyrosine, and tryptophan biosynthesis (IV = 1)	Phenylpyruvate L-Phenylalanine L-Tyrosine 3-(4-Hydroxyphenyl) pyruvate	0.00000 0.50000 0.50000 0.00000	Potential utility as biomarker for gastroesophageal and prostate cancer [24,25].	Phenylalanine and Tyrosine modulate neurotransmitter synthesis [26]. Tryptophan via the kynurenine pathway is critical for immune response [27].	IDO inhibitors have been explored for cancer therapies with varied results [28].
Histidine metabolism (IV = 0.598)	L-Histidine Carnosine Histamine N(pi)-Methyl-L-histidine N-Formimino-L-glutamate beta-Alanyl-N(pi)-methyl-L histidine Imidazole-4-acetate Methylimidazoleacetic acid L-Aspartate	0.22131 0.09016 0.18852 0.00000 0.04918 0.04918 0.00000 0.00000 0.00000	Histidine degradation can increase effectiveness of chemotherapy drugs.	Histidine metabolism may deplete Tetrahydrofolate (THF) reducing cancer cell DNA synthesis [29].	Histidine dietary supplementation for treatment of cancer.
Arginine biosynthesis (IV = 0.482)	L-Arginine N-Acetylornithine N-(L-Arginino) succinate L-Aspartate L-Citrulline L-Ornithine 2-Oxoglutarate Fumarate	0.07614 0.00000 0.11675 0.00000 0.22843 0.06091 0.00000 0.00000	Prognostic marker and predictor of survival in various cancers [30].	Arginine is involved in the biosynthesis of nitric oxide, agmatine, and polyamines and is associated with cell growth, invasion, and metastasis [31].	Arginine deprivation for anticancer therapies [32].
beta-Alanine metabolism (IV = 0.672)	beta-Alanine L-Aspartate Ureidopropionate 5,6-Dihydrouracil Carnosine beta-Alanyl-N(pi)-methyl-L-histidine L-Histidine Spermidine	0.39925 0.05597 0.10448 0.05597 0.05597 0.00000 0.00000 0.00000	β-alanine may elicit anti-tumor effects in breast cancer [33].	β-alanine reduces tumor cell migration and proliferation and increases breast cancer cell sensitivity to Doxorubicin [34].	β-alanine may have potential as a co-therapeutic agent for breast cancer.
Arginine and proline metabolism (IV = 0.653)	L-Arginine Putrescine Spermidine N-Acetylputrescine L-erythro-4-Hydroxyglutamate Hydroxyproline L-Proline L-Ornithine 4-Acetamidobutanoate	0.12442 0.18721 0.06628 0.04651 0.02674 0.02093 0.01744 0.16395 0.00000	Impacts cancer cell proliferation and survival.	Proline metabolism regulates reactive oxygen species and cytokine secretion [35]. Arginine modulates nitric oxide production.	Amino acid starvation therapies: PYCR1 and ProDH/Pox, and ASS1 as potential targets [36].
Phenylalanine metabolism (IV = 0.857)	L-Phenylalanine Phenethylamine Phenylpyruvate L-Tyrosine	0.35714 0.23810 0.26190 0.00000	Elevated phenylalanine may correlate with disease stage in ovarian cancer [37].	Phenylalanine hydroxylase converts phenylalanine to tyrosine influencing neurotransmitter synthesis.	Phenylalanine and tyrosine concentrations may have potential utility as biomarker targets.

^a^ Node importance calculated by the relative betweenness centrality. IV: impact value, the importance of each pathway, calculated as sum of matched metabolite nodes; thus, the maximum IV is 1.

## Data Availability

Data and supporting materials are not publicly available but may be available from the corresponding author upon reasonable request and approval of the investigators.

## Supplementary Materials

The following supporting information can be downloaded at: https://www.mdpi.com/article/10.3390/cancers17050891/s1, Figure S1: Box plots presenting the distribution of top metabolites that exhibited significant variation in all progressive disease (PD) group compared to the progression-free (PF) reference group.
