# Supplementary material for: Metabolomic Profiling of Disease Progression Following Radiotherapy for Breast Cancer
Source: Cancers (Basel). 2025 Mar 5;17(5):891. doi: 10.3390/cancers17050891 (PMC11899340; doi:10.3390/cancers17050891)
Supplement: Supplementary file 1 [file cancers-17-00891-s001.zip › cancers-3443731-supplementary.pdf]

**Supplementary Figure S1.** Box plots presenting the distribution of top metabolites that exhibited significant variation in all progressive disease (PD) group compared to the progression-free (PF) reference group.

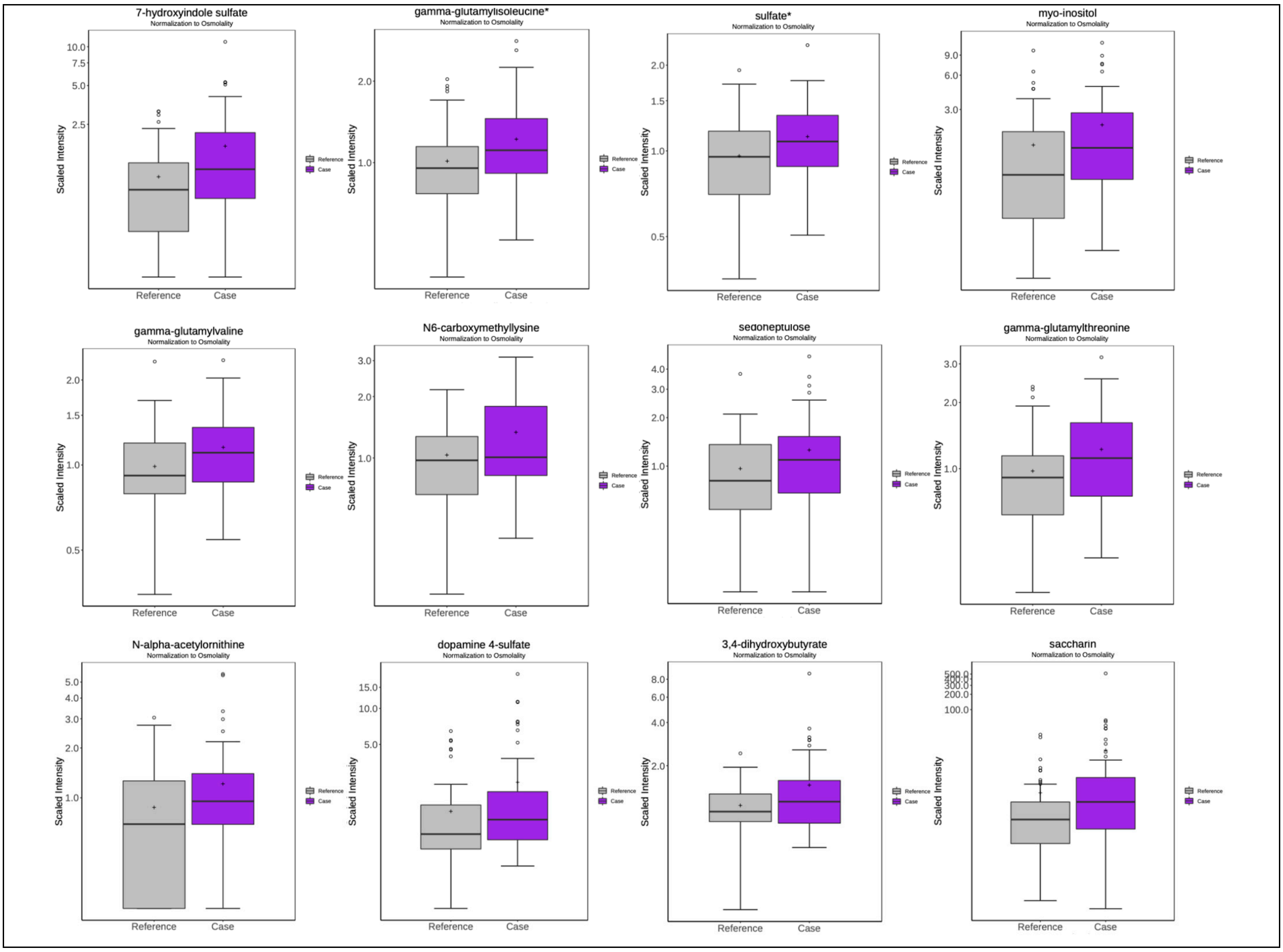


Note: The shaded boxes represent the middle 50% of the data (interquartile range, IQR), with whiskers indicating the full range within 1.5 times the IQR. The solid bar across each box represents the median, while the + symbol represents the mean. Data are scaled such that the median value was set to 1.0. Any outliers are displayed as individual dots beyond the whiskers.
